# Association Between Thromboelastography and Coagulation for Disease Severity Evaluation in Patients With Lower Extremity Arteriosclerosis Obliterans

**DOI:** 10.1002/jcla.25138

**Published:** 2024-12-23

**Authors:** Zhen Huang, Zibo Feng, Xiangli Bai, Xingxing Wang, Pengyun Wang, Liang Xiong

**Affiliations:** ^1^ Department of Laboratory Medicine, Liyuan Hospital, Tongji Medical College Huazhong University of Science and Technology Wuhan Hubei China; ^2^ Department of Vascular Surgery, Liyuan Hospital, Tongji Medical Collage Huazhong University of Science and Technology Wuhan Hubei China; ^3^ Department of Pathology Affiliated, Hangzhou Xixi Hospital Zhejiang University School of Chinese Medicine Hangzhou Zhejiang China

**Keywords:** blood coagulation, elderly patient, lower extremity arteriosclerosis obliterans, surgery, thrombelastography

## Abstract

**Background:**

Thromboelastography (TEG) and coagulation tests can be used to detect hypercoagulability to assess thrombus formation. This study explored the association between TEG and coagulation in evaluating disease severity in elderly patients with lower extremity arteriosclerotic occlusive disease (LEASO), aimed to provide surgical treatment guideline.

**Methods:**

We retrospectively analyzed the clinical characteristics, laboratory biomarkers, TEG and coagulation parameters of 233 elderly LEASO patients treated between 2020 and 2023. Among them, 86 underwent surgical amputation, 51 received vascular intervention, and the remaining were treated conservatively. Differences in TEG and coagulation among the three groups were assessed using Spearman's correlation. Multivariate logistic regression and receiver operating characteristic curves analyzed the relationships among TEG, fibrinogen (FIB), and D‐dimer (D‐D) levels for surgical evaluation.

**Results:**

Inflammatory factors, platelet counts, and Fontaine stages III‐IV differed significantly between the surgery and conservative groups (*p* < 0.05). The surgery group had higher α‐angle, maximum amplitude (MA), coagulation comprehensive index (CI), FIB, and D‐D, whereas lower clotting time (*K*) compared to the conservative group (*p* < 0.05), correlated with a lower ankle brachial index (ABI), indicating more severe clinical presentation. Spearman's analysis identified positive associations between α‐angle, MA with FIB and D‐D levels in surgical patients. Area under curve analysis indicated that combining MA, α‐angle, FIB, and D‐D could enhance accuracy in evaluating surgical necessity in LEASO.

**Conclusion:**

In elderly LEASO patients, TEG and coagulation analysis revealed a positive association between thrombus intensity and disease severity. Increased MA, α‐angle, FIB, and D‐D levels serve as predictors for surgical treatment necessity in LEASO.

## Graphical abstract

This study confirmed a significant correlation between TEG and coagulation function. Elevated FIB and D‐D levels combine with increased MA and α‐angle of TEG to determine systemic hypercoagulation status and thrombosis risks. The application of TEG and coagulation indices provides a more rapid and intuitive supplementary means for early monitoring of LEASO, reflects the degree of lesions, and provides a new perspective for evaluating surgical treatment.

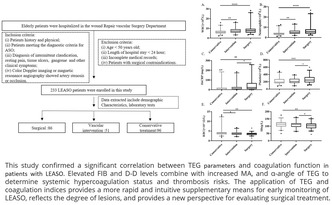

## 1 Introduction

Arteriosclerosis obliterans (ASO) is characterized by atherosclerotic thickening, loss of elasticity, and medial calcification of the arterial wall, which lead to inadequate blood flow to the lower extremities and sclerotic narrowing or blockage of the arteries in the legs [1, 2]. Lower extremity arteriosclerosis obliterans (LEASO) is one of the most common peripheral arterial diseases (PADs) and is characterized by lower extremity ischemic symptoms [3]. The prevalence of ASO is increasing annually in China and Western countries; ASO affects up to 45.3 million people in China, and approximately 30% of such individuals are over 50 years of age. Critical limb ischemia (CLI) is the most severe manifestation of the disease, and delayed diagnosis and treatment can result in limb loss or even death. For patients with CLI, arterial revascularization is an imperative treatment for salvaging the ischemic limbs [4, 5, 6].

The clinical symptoms of ASO can be categorized into four stages: Fontaine stage I (no or atypical symptoms, swollen and painful limbs), stage II (intermittent claudication), stage III (rest pain), and or stage IV (non‐healing ulcers and gangrene) [7, 8]. LEASO is associated with various risk factors, including advanced age, diabetes mellitus, hyperlipidemia, hypertension, and cigarette smoking. In addition to having significantly reduced physical function and quality of life, patients with LEASO have a higher risk of cardiovascular events such as stroke and myocardial infarction [9, 10]. Medication, conventional surgery, and percutaneous endovascular therapy are the therapeutic methods used in LEASO [11]. Drug therapy in Western medicine involves anticoagulation, thrombolysis, tube expansion, and lipid‐lowering. Endovascular interventional therapy has gradually attracted acceptance as a simple procedure with minimal trauma [12]. Some patients require limb amputation due to severe limb necrosis and infections, severe intermittent claudication, rest pain, ischemic gangrene, and long‐term nonhealing ischemic ulcers. Certain patients may have lower extremity artery disease with stenosis and occlusion that cannot be adequately treated. LEASO‐induced limb ischemia considerably worsens patient prognosis and increases disability and mortality rates [13, 14].

Coagulation assessment is particularly important in patients with trauma, spinal joint replacement, and surgical treatment owing to blood loss and coagulopathy [15]. Clinically, surgeons use thromboelastography (TEG) to provide information about a patient's coagulation status by assessing thrombosis and lysis. TEG is widely used to diagnose coagulopathies, monitor bleeding, evaluate hemostasis, and guide transfusion in patients with trauma or those undergoing surgery [16]. Furthermore, TEG is a viscoelastic test that dynamically monitors the coagulation reaction, from fibrin formation to clot dissolution, and provides information such as clotting time (*K*), clot formation rate, maximum clot strength, and degree of fibrinolysis [8, 17]. By analyzing these parameters, trauma physicians can diagnose a patient's condition better, faster, and more accurately and prescribe appropriate treatments. In PADs, TEG has been used to predict disease severity and analyze the impact of contrast on coagulation parameters and is currently the only diagnostic modality that is useful for detecting hypercoagulable states [18].

At present, no comprehensive reports on the use of TEG and coagulation assays to evaluate LEASO severity have been published. We aimed to address this research gap by collecting clinical data from 233 patients with LEASO. Analyzed the association between LEASO severity and coagulation/TEG parameters to provide clinical treatment guidelines and prognostic monitoring insights.

## 2 Materials and Methods

### 2.1 Study Design and Participants

We conducted an open retrospective study and collected wound repair and vascular surgery results over the past 2 years from the clinical laboratory information system. Elderly patients hospitalized between June 2020 and January 2023 in the Wound Repair and Vascular Surgery Departments of Liyuan Hospital, affiliated with Tongji Medical College, Huazhong University of Science and Technology, were selected as research participants. Patients with LEASO symptoms that met the following criteria were included: (i) patients history and physical examination; (ii) patients meeting the diagnostic criteria for LEASO [2]; (iii) diagnosis of intermittent claudication, resting pain, tissue ulceration, gangrene, and other clinical symptoms; and (iv) color Doppler imaging or magnetic resonance angiography revealing artery stenosis or occlusion [19]. Patients who met the following criteria were excluded: (i) age < 50 years old; (ii) hospital stay < 24 h; (iii) incomplete medical records; and (iv) surgical contraindications. Finally, 233 patients with LEASO patients, with a mean age of 69.29 ± 11.50 years, were enrolled in the study (141 males and 92 females). Based on hospitalization records, 96 patients received conservative treatment (exercise or medication), and the remaining patients were treated with revascularization, of whom 51 underwent vascular intervention (angiography or balloon angioplasty) and 86 underwent open repair surgery (arterial bypass or amputation).

### 2.2 Study Measurement

Patient demographic information, including age, sex, disease duration, initial clinical symptoms, disease severity classification, and comorbidities, were extracted from the hospital database. The ankle brachial index (ABI) was measured during hospitalization in the vascular surgery ward, with the recorded ABI being the lower value between the left and right legs. Peripheral venous blood samples were collected from each patient for the analysis of coagulation function, TEG, blood cell counts (Mindray, China, CAL‐8000), liver and kidney biochemical functions, levels of the inflammatory biomarker high‐sensitivity C‐reactive protein (hs‐CRP) (Beckman, Coulter‐AU5800), and myocardial function (Roche, Germany, Cobas‐6000) at our hospital's clinical laboratory. Plasma hs‐CRP levels were measured using enzyme‐linked immunosorbent assay. Disease severity was categorized into Fontaine stages I–IV based on the clinical manifestations. Other indicators used to evaluate severity included preoperative or postoperative ABI and admission rates for prognostic assessment within half a year. The readmission rate only counted the patients who needed to be readmitted to the hospital due to post‐treatment infection, disease recurrence, postoperative re‐occlusion, ischemia–reperfusion injury, and drug monitoring.

### 2.3 Thromboelastography (TEG) Detection

Thromboelastography (TEG) detection was performed at CFMS (China, LEPU‐8800) in our hospital. The operating instructions were as follows: 1 mL of sodium citrate‐anticoagulated whole blood was transferred into a tube containing the reagent (0.3 mg/mL kaolin + normal saline), mixed thoroughly, and allowed to stand for 3–5 min to activate the blood. Simultaneously, 20 μL of reagent 2 (CaCl_2_) was pipetted into the standard cup, followed by adding 340 μL of whole blood. The cup was positioned in the “test” position, and detection was completed in approximately 30 min.

Among the TEG parameters, coagulation factor activity (*R*) reflects the comprehensive effect of factors involved in the coagulation initiation process. The clotting time (*K*) is the time from measurement of *R* (blood begins to coagulate quickly) to the time of blood clot hardness reaching a fixed level (amplitude = 20 mm) [20]. The α‐angle is taken from the beginning of coagulation, and the measurement indicates the α‐angle between the baseline and tangent of the TEG curve, and shows the acceleration and kinetics of fibrin formation and cross‐linking. The maximum amplitude (MA) directly measures the highest point on the TEG curve and represents clot strength. The MA depends on platelet (PLT) concentration, PLT function, and PLT‐fibrin interactions [21]. The coagulation comprehensive index (CI) is the total clot strength, describes all coagulation interactions and reflects the patient's overall coagulation. The formula for calculating the CI is 0.1227 *R* + 0.0092 *K* + 0.1655MA–0.0241α‐angle–5.0220 [22].

### 2.4 Detection of Blood Coagulation

In vitro anticoagulated whole blood was centrifuged at 4000 rpm for 6 min to separate the plasma. The CS‐5100 automatic blood coagulation analyzer (Sysmex, Japan) was used to measure prothrombin time (PT), activated partial thromboplastin time (APTT), thrombin time (TT), fibrinogen (FIB), D‐dimer (D‐D), and prothrombin time‐international normalized ratio (PT‐INR).

### 2.5 Statistical Analysis

Statistical analyses were conducted using the SPSS statistical software (version 26.0; Chicago, IL, USA) and GraphPad Prism software (version 7.0; San Diego, CA, USA). Measurement data were expressed as mean ± SD ($\bar{x}$±s), abnormal distribution was showed by interquartile range, chi‐square ($\chi^{2}$) test was used for categorical variables, and the Kolmogorov–Smirnov test was performed to calculate the distribution of different variables. Variables with a non‐normal distribution were expressed as geometric means (95% confidence intervals), and count data were expressed as percentages (%). Multiple comparison analysis of variance (ANOVA) with pairwise comparisons was used to identify significant effects, and Spearman's correlation analysis was used to analyze the correlation between TEG and coagulation. The area under the receiver operating characteristic (ROC; AUC) curve was used to predict model performance. All statistical tests were two‐tailed, and *p* < 0.05 indicated statistical significance.

## 3 Results

### 3.1 Demographic and Characteristics of Patients With LEASO

**FIGURE 1:**
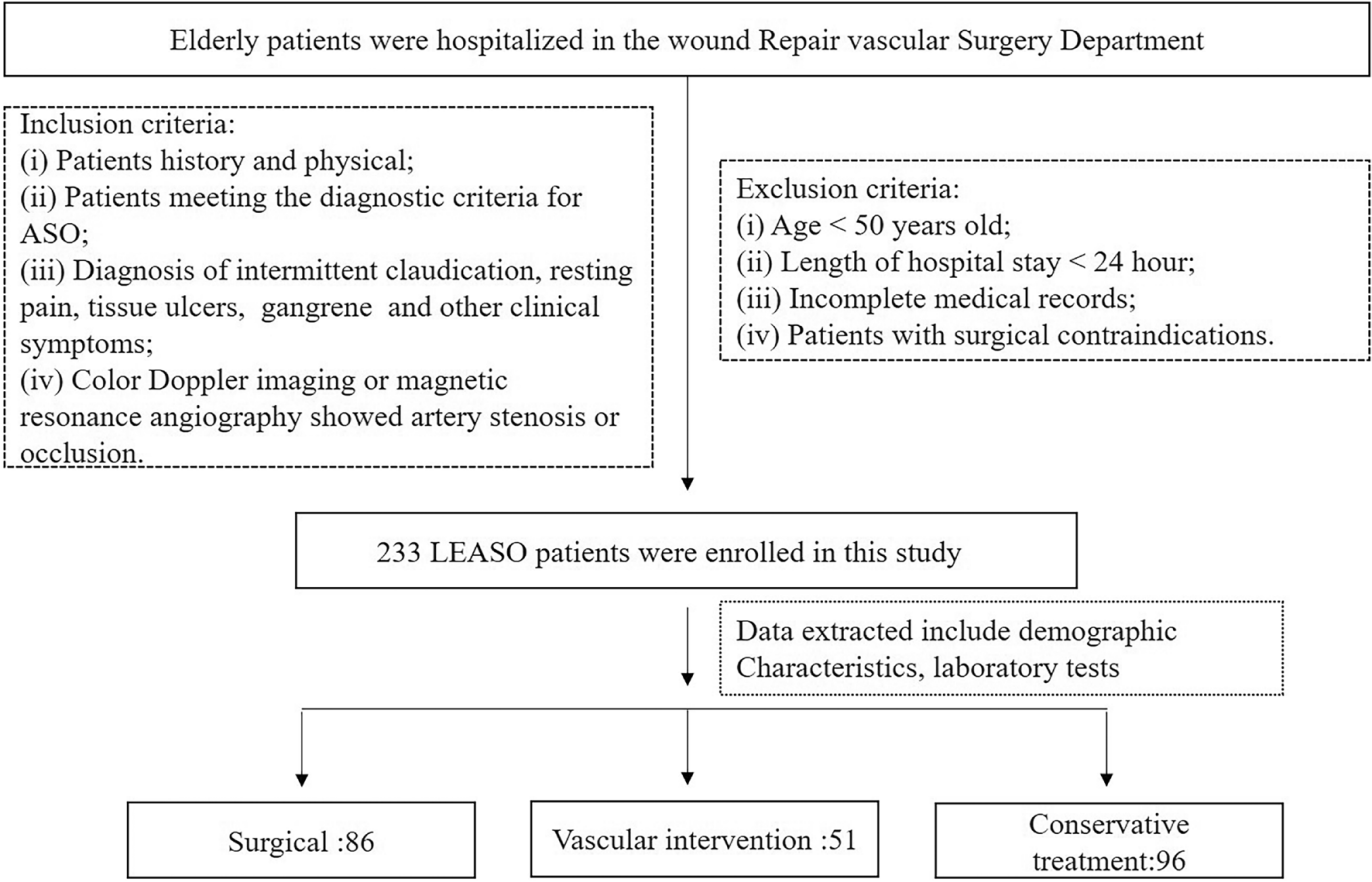
Flowchart for patient enrollment. LEASO, lower extremity arteriosclerosis obliterans.

Based on medical records and treatment methods, patients with LEASO were categorized into three groups. Eighty‐six patients underwent surgical therapy, including arterial bypass or amputation and had a mean age of 68.65 ± 10.13 years (51 males and 35 females), 51 patients received vascular intervention therapy (balloon dilatation or stent implantation) and had a mean age of 70.61 ± 10.06 years (31 males and 20 females), and the remaining 96 patients received conservative treatment (exercise or general drug therapy) and had a mean age of 69.133 ± 13.28 years (59 males and 37 females; Figure 1).

**FIGURE 2:**
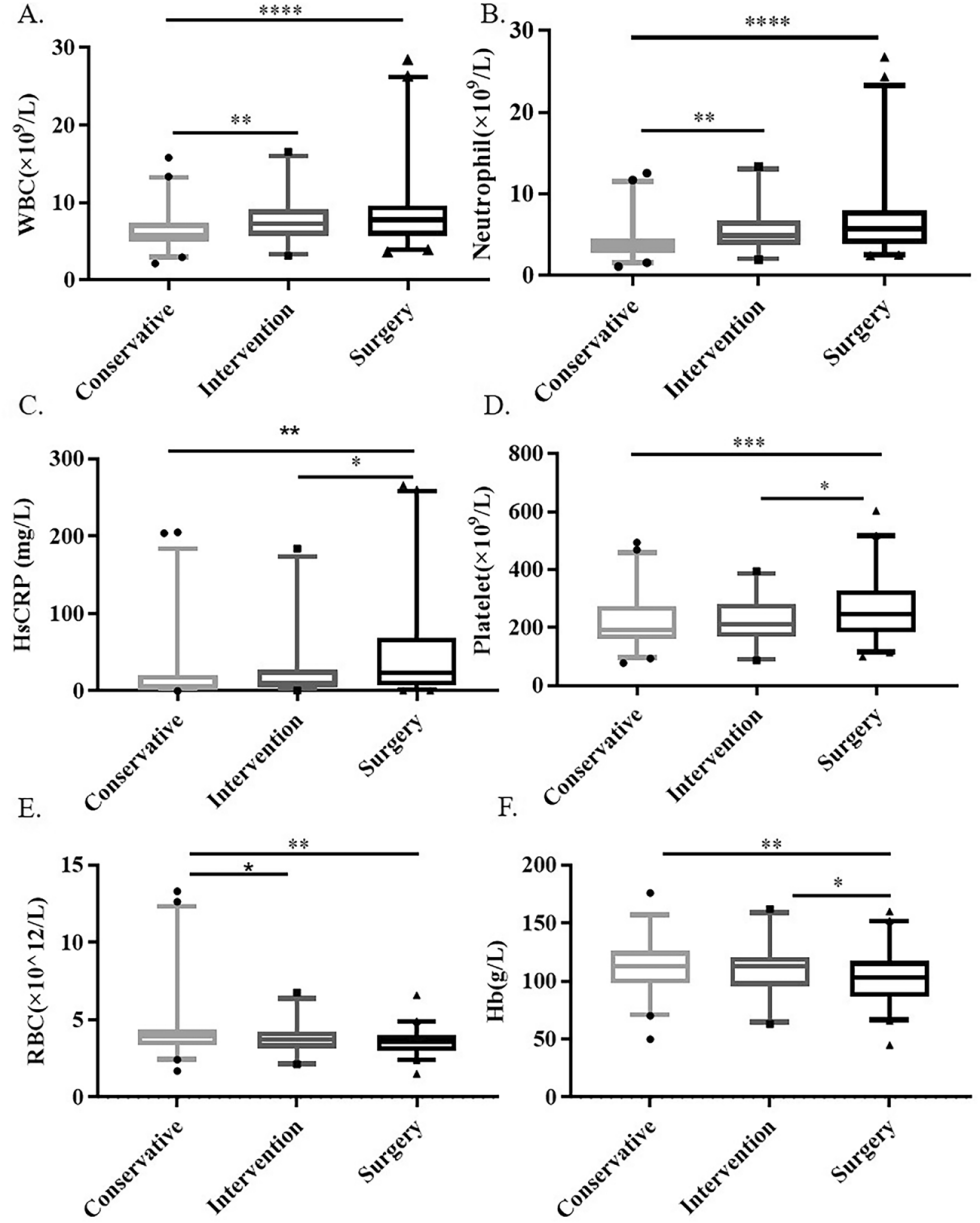
Comparison of WBC, neutrophil, hs‐CRP levels, PLT, RBC, Hb concentration among the three groups. (A) Comparison of WBC counts among groups; (B) Comparison of neutrophil counts among groups; (C) Comparison of hs‐CRP levels among groups; (D) Comparison of PLT counts among groups; (E) Comparison of RBC counts among groups; (F) Comparison of Hb concentration among groups. Hb, hemoglobin; hs‐CRP, high‐sensitivity C‐reactive protein; PLT, platelet; RBC, red blood cells; WBC, white blood cells. *p < 0.05; **p < 0.01; ***p < 0.001; **** *p* < 0.0001.

We retrospectively analyzed the basic demographic characteristics of the three groups and found no significant intergroup differences in age, sex, ration of males‐to‐females, or variations in laboratory indices related to liver, kidney, and heart function. No statistically significant differences were noted in the prevalence of coronary heart disease among the groups (*p* > 0.05). However, the surgery group had a higher complication rate of hypertension and diabetes, longer hospitalization duration, and a greater proportion of patients classified under Fontaine stages III–IV compared to the conservative group (Table 1). Additionally, the mean levels of red blood cells (RBC) and hemoglobin (Hb) in the surgery group were slightly lower than those in the conservative group, and the RBC and Hb values in the three groups were within the reference range for healthy people (*p* < 0.05). However, white blood cells (WBC), neutrophil, and platelet (PLT) counts and high‐sensitivity C‐reactive protein (hs‐CRP) levels more varied significantly between the surgery and conservative groups than between the intervention and conservative groups (*p* < 0.05), and hs‐CRP is associated with inflammation activity. The surgery group also showed higher levels of inflammatory factors (Figure 2).

**TABLE 1:** Demographic and clinical characteristics of patients in the three groups.

Variables	Conservative (*n* = 96)	Intervention (*n* = 51)	Surgery (*n* = 86)	*p*‐value
Gender				
Male, *n* (%)	59 (61.46)	31 (60.78)	51 (59.30)	0.956
Female, *n* (%)	37 (38.54)	20 (39.22)	35 (41.70)	
Age (years)	69.13 ± 13.28	70.61 ± 10.06	68.65 ± 10.13	0.277
Hospital stays (days)	13.79 ± 6.80^c^	15.33 ± 7.44^b^	20.51 ± 7.35	0.009
Laboratory characteristics				
ALT (U/L)	18.92 ± 18.01	17.96 ± 13.43	17.68 ± 13.91	0.424
AST (U/L)	22.85 ± 16.60	21.42 ± 12.96	23.20 ± 15.12	0.415
TNT‐t (μg/L)	58.19 ± 173.53	43.87 ± 65.17	64.31 ± 156.87	0.495
Cr (μmol/L)	85.17 ± 85.17	105.70 ± 109.55	128.00 ± 149.39	0.437
Fontaine stage, *n* (%)				
I	58 (75.94)^a^, ^c^	14 (61.22)	24 (45.79)	0.001
II	29 (17.72)	21 (18.37)	26 (18.07)	
III	6 (5.06)	9 (8.17)	18 (15.66)	
IV	3 (1.28)	6 (12.24)	17 (20.48)	
Complications, *n* (%)				
Hypertension	46 (47.92)^a^, ^c^	37 (72.55)	58 (67.44)	0.004
CAD	25 (26.04)	17 (33.33)	29 (33.72)	0.469
Diabetes	60 (62.50)^c^	41 (80.39)	73 (84.88)	0.001

*Note:* Data are presented as the mean ± SD of categorical variables.

Abbreviation: CAD, coronary artery disease.

^a^ *p* < 0.05 between conservative and intervention groups.

^b^ *p* < 0.05 between intervention and surgery groups.

^c^ *p* < 0.05 between conservative and surgery groups.

### 3.2 Comparison of TEG and Coagulation Parameters in the Surgery, Intervention, and Conservative Groups

The mean α‐angle, MA, and CI values of patients in the surgery group were 72.75°, 72.50 mm, and 2.50, respectively. In comparison, those in the intervention group were 69.16°, 68.88 mm, and 1.08, respectively, and whereas in the conservative group were 65.04°, 62.11 mm, and 0.11, respectively. The α‐angle, MA, and CI values were significantly higher in the surgery and intervention groups compared to that in the conservative group (*p* < 0.001). The mean *K* in each group was 1.18, 1.56, and 1.80 min, respectively (*p* < 0.001), and was significantly lower in the surgery group compared to the conservative group (*p* < 0.05). The levels of the coagulation parameters FIB (5.44 g/L) and D‐D (1.43 mg/L) were significantly higher in the surgery group than those of FIB (3.55 g/L) and D‐D (0.79 mg/L) in the conservative group. Elevated fibrinogen levels in patients with PADs have been found to increase the risk of adverse outcomes [23]. The differences among the three groups were statistically significant, and more statistically significant differences were observed in the TEG parameters and FIB and D‐D coagulation indices between the surgery group and conservative group than those between the conservative group and the intervention group (Table 2).

**TABLE 2:** Comparison of TEG and coagulation parameters of patients in the three groups.

Factor	Conservation *n* = 96	Intervention *n* = 51	Surgery *n* = 86	*F*	*p* value
*K* (min)	1.80 [1.64–1.96]^c^	1.56 [1.36–1.75]^b^	1.18 [1.10–1.25]	21.000	< 0.001
α‐angle	65.04 [63.38–66.69]^a^, ^c^	69.16 [67.19–71.12]^b^	72.75 [71.61–73.90]	27.807	0.001
MA (mm)	62.11 [60.69–63.52]^a^, ^c^	68.88 [66.84–70.92]^b^	72.50 [71.12–73.89]	53.878	< 0.001
CI	0.11 [−0.38–0.60]^a^, ^c^	1.08 [0.15–2.02]^b^	2.50 [2.11–2.90]	21.603	< 0.001
*R* (min)	6.01 [5.65–6.38]	6.02 [5.27–6.77]	5.53 [5.17–5.89]	1.664	0.192
PT (s)	12.25[11.87–12.62]	13.13 [11.35–14.90]	12.73 [12.40–13.06]	1.270	0.283
APTT (s)	27.75 [27.00–28.51]	29.27 [27.45–31.09]	28.73 [27.79–29.67]	2.006	0.137
TT (s)	17.58 [17.24–17.92]	17.44 [16.63–18.25]	17.47 [16.96–17.98]	0.090	0.914
FIB (g/L)	3.55 [3.34–3.75]^a^, ^c^	4.18 [3.77–4.58]^b^	5.44 [5.04–5.83]	35.137	< 0.001
INR	1.25 [0.90–1.61]	1.15 [0.98–1.32]	1.12 [1.09–1.15]	0.317	0.729
D‐D (mg/L)	0.79 [0.68–0.90]^a^, ^c^	1.30 [0.97–1.62]	1.43 [1.14–1.72]	8.881	< 0.001

Multiple comparison (mg/L)		*K* (min)	a‐angle	MA (mm)	FIB (g/L)	D‐D
*p* ^a^	C vs. I	0.170	0.005	0.000	0.021	0.013
*p* ^b^	I vs. S	0.002	0.002	0.003	0.000	0.913
*p* ^c^	C vs. S	0.000	0.001	0.000	0.000	0.000

*Note:* Data are means with 95% CI (confidence intervals). *p* < 0.05 overall comparison.

Abbreviations: APTT, activated partial thromboplastin time; CI, coagulation comprehensive index; D‐D, D‐dimer; FIB, fibrinogen; INR, national standardized ratio; *K*, blood clot formation time; MA, maximum amplitude; PT, prothrombin time; *R*, coagulation factor activity; TEG, thromboelastography; TT, thrombin time; α‐angle, blood coagulation velocity.

^a^ *p* < 0.05 between conservative and intervention groups.

^b^ *p* < 0.05 between intervention and surgery groups.

^c^ *p* < 0.05 between conservative and surgery groups.

### 3.3 Correlation Analysis Between TEG and Coagulation Parameters in the Surgery Group

A correlation analysis of nonparametric tests between TEG and blood coagulation parameters in the surgery group was performed using the Spearman's rank test. The α‐angle correlated positively with PT, FIB, and D‐D: *r*_s_ = 0.221, *r*_s_ = 0.254, *r*_s_ = 0.215; MA correlated positively with PT, FIB, and D‐D: *r*_s_ = 0.287, *r*_s_ = 0.323, *r*_s_ = 0.222; the CI correlated positively with FIB and D‐D: *r*_s_ = 0.248, *r*_s_ = 0.392; and R correlated negatively with D‐D: *r*_s_ = −0.366. The most significant correlations were observed between FIB, D‐D of coagulation, and α‐angle, MA, Cl of TEG, with statistical significance, PT was positively correlated with α‐angle and MA, indicating that the combination of PT and TEG could reflect exogenous coagulation state (*p* < 0.05; Table 3).

**TABLE 3:** Correlation analysis between TEG and coagulation in the surgery group.

	PT		APTT		TT		FIB		D‐D	
	*r*_s_	*p*	*r*_s_	*p*	*r*_s_	*p*	*r*_s_	*p*	*r*_s_	*p*
*K*	−0.174	0.109	0.017	0.878	0.049	0.653	−0.190	0.080	−0.211	0.051
α‐angle	0.221*	0.041	0.011	0.923	−0.063	0.567	0.254*	0.018	0.215*	0.047
MA	0.287*	0.007	0.144	0.188	−0.032	0.772	0.323**	0.002	0.222*	0.040
CI	0.120	0.271	−0.157	0.151	−0.122	0.265	0.248*	0.022	0.392**	0.000
*R*	0.068	0.532	0.326**	0.002	0.099	0.365	−0.068	0.535	−0.366*	0.001

Abbreviations: APTT, activated partial prothrombin time; CI, coagulation index; D‐D, d‐dimer; FIB, fibrinogen; *K*, blood clot formation time; MA, maximum amplitude; R, coagulation factor activity; TEG, thromboelastography; TT, thromboplastin time; α‐angle, blood coagulation velocity.

* *p* < 0.05.

** *p* < 0.01.

### 3.4 Evaluation of Disease Severity and Prognosis in Patients With LEASO

The ABI values of the preoperative intervention and surgery groups were 0.59 and 0.42, respectively, which were significantly lower than that of the conservative group, which was 0.85. After the multiple comparison analysis, significant differences were identified in preoperative ABI index among three groups (*p* < 0.01). The ABI of the intervention and surgery group after treatment improved to 0.81 and 0.70, respectively, with a significant increase from pretreatment levels (*p* < 0.001). Additionally, the half‐year readmission rates of the conservative, intervention, and surgery groups were 22.92%, 50.98%, and 55.81%, respectively, and the readmission rate due to post‐treatment infection, disease recurrence, postoperative re‐occlusion, ischemia–reperfusion injury, and drug monitoring was significantly higher in the surgery group than in the other two groups (Table 4).

**TABLE 4:** Comparison levels of ABI and readmission rate among three groups.

Factor	Conservative	Intervention	Surgery	*F*	*p*
	*n* = 96	*n* = 51	*n* = 86		
Preoperative ABI	0.85 [0.78–0.93]^a^, ^c^	0.59 [0.50–0.68]^b^	0.42 [0.37–0.46]	45.696	< 0.001
Preoperative ABI	None	0.81 [0.64–0.78]	0.70 [0.63–0.78]	3.069	0.082
*t*		−3.674	−6.597		
*p*		< 0.001	< 0.001		
Half‐year readmission	22 (22.92%)^a^, ^c^	26 (50.98%)	48 (55.81%)	14.365	0.002

*Note:* Data are means with 95% CI (confidence intervals). *p* < 0.05 overall comparison.

Abbreviation: ABI, ankle‐brachial index.

^a^ *p* < 0.05 between conservative and intervention groups.

^b^ *p* < 0.05 between intervention and surgery groups.

^c^ *p* < 0.05 between conservative and surgery groups.

### 3.5 Multivariate Logistic Analysis of Surgical Treatment in Patients With LEASO

The univariate logistic regression analysis revealed that elevated MA, α‐angle, FIB, D‐D, and PLT were the main determinants of LEASO treatment. The analysis revealed that the MA (OR = 1.138, *p* = 0.002), α‐angle (OR = 1.088, *p* = 0.042), FIB (OR = 1.696, *p* = 0.002), D‐D (OR = 2.224, *p* = 0.032), and PLT (OR = 1.006, *p* = 0.05) with the surgical treatment in patients with LEASO, Diabetes was found to be an independent risk factor for LEASO (Table 5).

**TABLE 5:** Multivariate logistic analysis of patients treated with surgery.

Variable	Univariate analysis			
	*β*	Ward	OR (95% CI)	*p*
MA	0.130	9.694	1.138 (1.049, 1.235)	0.002
α‐angle	0.084	4.428	1.088 (1.003, 1.179)	0.042
FIB	0.528	10.071	1.696 (1.224, 2.350)	0.002
D‐D	0.799	4.585	2.224 (1.070, 4.623)	0.032
hs‐CRP	0.004	5.339	1.004 (0.993, 1.016)	0.468
PLT	0.005	7.871	1.006 (1.002, 1.010)	0.005
Diabetes	−1.401	5.952	0.246 (0.080, 0.759)	0.015

Abbreviations: CI, confidence interval; D‐D, D‐dimer; FIB, fibrinogen; hs‐CRP, high‐sensitivity C‐reactive protein; MA, maximum amplitude; OR, odds ratio; PLT, platelet; α‐angle, blood coagulation viscosity.

### 3.6 ROC Curve Analysis for TEG and Coagulation in Predicting Surgical Treatment in LEASO Patients

**FIGURE 3:**
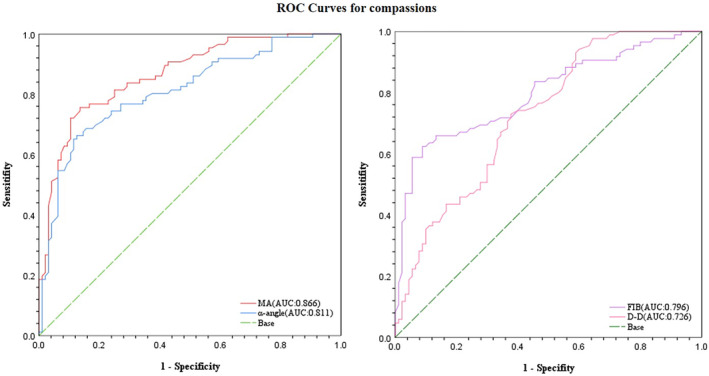
Predictive performance of models; ROC curves for TEG and coagulation to predict surgical treatment in LEASO patients. (A) MA and α‐angle; (B) FIB and D‐D. D‐D, D‐dimer; FIB, fibrinogen; LEASO, lower extremity arteriosclerosis obliterans; MA, maximum amplitude; ROC, receiver operating characteristic curve; α‐angle, blood coagulation viscosity.

The ROC curve analysis suggested optimal cutoff points for TEG and coagulation parameters in determining surgical amputation in LEASO patients. The identified cutoff values for MA and α‐angle were 69.35 mm, 72.35°, respectively, and AUC values were 0.866 and 0.811, respectively. Additionally, the cutoff values for FIB and D‐D were 4.58 g/L and 0.76 mg/L, with AUC values of 0.796 and 0.726, respectively. Notably, when the α‐angle, MA, and FIB levels are beyond the cutoff values, the formation rate of blood clot accelerates, the intensity of blood clot increases, and the body tends to be in a hypercoagulable state, which had a higher specificity to evaluate the surgical treatment of LEASO patients (*p* < 0.001; Table 6 and Figure 3).

**TABLE 6:** ROC curve results of FIB and TEG parameters in predicting disease severity in patients with LEASO.

Model	AUC	Cut‐off	Sensitivity (%)	Specificity (%)	Youden index
MA (mm)	0.866 (0.814, 0.918)	69.35	75.6	86.5	0.620
α‐angle (°)	0.811 (0.747, 0.874)	72.35	65.1	88.5	0.537
FIB (g/L)	0.796 (0.729, 0.863)	4.58	62.4	91.1	0.535
D‐D (mg/L)	0.726 (0.653, 0.800)	0.76	72.9	62.2	0.352

Abbreviations: AUC, area under curve; D‐D, D‐dimer; FIB, fibrinogen; MA, maximum amplitude; ROC, receiver operating characteristic curve; α‐angle, blood coagulation viscosity.

## 4 Discussion

Peripheral arterial disease (PAD) is a chronic atherosclerotic process that causes narrowing of the peripheral arterial vasculature, predominantly in the lower limbs [6]. Most patients with PAD are old and present with multiple organ dysfunction, whereas those with diabetes and LEASO tend to have more severe ischemic symptoms [24]. The Fontaine classification is commonly used to stage ischemia in the extremities, with ischemic resting pain indicating an advanced stage of the disease. Clinically, the treatment selection for LEASO depends on the assessment of disease severity and prognosis. In patients with critical limb ischemia (CLI), vascular intervention or surgical treatment should be promptly performed [25, 26]. If symptoms are severe, surgery should be performed as soon as possible to restore blood flow through the occluded vessels, alleviate lower limb ischemic symptoms, and prevent distal necrosis.

This study found high incidence of complications of diabetes and hypertension in the surgery group; thus, patients with PAD complicated by diabetes are more likely to have arterial lesions [27]. Compared with the other groups, the surgery group showed significantly elevated WBC, neutrophils, PLT counts, and hs‐CRP levels (*p* < 0.05). Inflammation markers levels substantially increased in the surgery group; however, the PLT counts did not assess platelet function. Blood analysis can determine systemic hypercoagulable or hypocoagulable states to help determine the risk of thrombosis and bleeding, and can assist clinicians in predicting the likelihood of severe diseases such as ulcers and gangrene in patients with LEASO. Notably, this study collected TEG data in conjunction with coagulation parameters for a comprehensive analysis, providing a method for evaluating the severity of LEASO.

Most conventional coagulation tests, such as prothrombin time/international normalized ratio (PT/INR) and activated partial thromboplastin time (APTT), these assays are limited to the initiation phase of clot formation [28]. PT/INR is affected only by intrinsic pathways and fibrinogen and does not account for role of anticoagulant pathways, platelets, or endothelial cells [29]. PT is a screening test for detection of exogenous coagulation factor [30]. Previous studies have reported that the levels of FIB, D‐D antigens, von Willebrand factor, and tissue plasminogen activators are higher in patients with PAD than in healthy individuals [31, 32], and certain coagulation factors can serve as markers of PAD progression [33]. In the current study, FIB and D‐D levels were significantly higher in the surgery group than the conservative group among patients with LEASO (*p* < 0.05). FIB, which is both a coagulation factor and an acute phase response marker of inflammation, is a component of atherosclerotic plaques. Furthermore, elevated FIB levels predict PAD severity, indicate future disease progression, and increase the risk of adverse outcomes [23, 34]. Circulating D‐D levels depend on FIB formation and fibrinolytic activity, and elevated D‐D levels correlate with ABI and can serve as a short‐term predictor of all‐cause mortality in patients with PAD [35, 36]. Although D‐D and FIB can serve as specific indicators of hypercoagulability, they cannot fully represent the coagulation function of systemic blood [37, 38].

Compared with conventional coagulation assays, TEG accurately evaluates the coagulation state in whole‐body blood [39]. Continuous viscoelastic changes associated with fibrin indicators have been recorded from the initial thrombin generation to fibrin strand formation to fibrinolysis [40]. Trauma resuscitation is currently gaining attention in clinical practice. Our study showed that patients in the surgery group displayed a hypercoagulable state prior to surgery, as indicated by TEG parameters: decreased time to reach clot strength (*K*), increased rate of clot formation (α‐angle), and enhanced clot strength (MA). The *K* indicates the speed of blood‐clot netting, while the α‐angle indicates clotting rate primarily influenced by PLT, thrombin, and FIB function. The α‐angle closely relates to *K*, and both parameters serve as indicators of FIB function and reflecting the clot aggregation rate [41, 42]. The increased α‐angle indicates increased fibrin generation due to enzymatic hypercoagulability. MA represents the maximum platelet–fibrin clot strength and is influenced by changes in fibrinogen, platelet count, and function. Previous studies have indicated the criteria of high‐level clot strength as “MA_thrombin_ ≥ 68 mm,” based on ROC curve analysis for predicting clinical outcomes [18, 43, 44]. TEG can be used to detect and evaluate the whole picture of coagulation in terms of coagulation factors, FIB, platelet function, and fibrinolysis.

The ABI decreased with a reduction in the arterial blood supply to the lower extremities, and this decrease positively correlated with the degree of ischemia in the lower extremities. The ABI has been widely used for the early diagnosis, condition evaluation, intraoperative examination, and treatment effect follow‐up of LEASO [45]. In clinical practice, ABI ≤ 0.90 can identify patients with PAD with serious stenosis (> 50%); however, this cutoff may not be sufficiently sensitive to represent disease severity [3, 46]. Our study suggested that the average preoperative ABI of 0.42 in the surgery group was significantly lower than the preoperative ABI of 0.85 in the conservative group. Compared with the other groups, the surgery group had a higher proportion of patients with Fontaine stages III–IV and more severe clinical symptoms, thereby affecting their quality of life. Following revascularization, a significant improvement was observed in postoperative ABI values; however, patients with arterial occlusion need to be readmitted for treatment due to post‐treatment infection, disease recurrence, postoperative re‐occlusion, ischemia reperfusion injury, and other problems. Statistical data showed that the surgery group exhibited a higher half‐year admission rate than the other two groups.

In this study, the Spearman's test was used to analyze the correlation between TEG and coagulation in the surgery group. The α‐angle positively correlated with FIB and D‐D levels, with coefficients of 0.254 and 0.215, respectively. Similarly, MA positively correlated with FIB and D‐D levels, with coefficients of 0.323 and 0.222, respectively, as did CI, with coefficients of 0.248 and 0.392, respectively (*p* < 0.05). Among the TEG parameters, the correlation coefficients between MA, α‐angle, CI, FIB, and D‐D were more significant. The FIB and D‐D were positively correlated with a‐angle and MA showed that with the elevation of FIB and D‐D levels, the formation rate of blood clot accelerated, the strength or hardness of blood clot increased, and the body tended to the hypercoagulable state. Relevant studies have confirmed that these TEG parameters are associated with FIB, and that D‐D can robustly predict the coagulation status of patients from all clinical aspects [47]. Univariate logistic regression analysis revealed that the MA, α‐angle, PLT, FIB, and D‐D associated with surgery in patients with LEASO, reflected the correlation between fibrinogen level and function, PLT and the speed of blood clot formation, and the strength of blood clot. Areas under the ROC curves for MA and α‐angle were 0.866 and 0.811 (*p* < 0.001), the optimal cutoff values were 69.35 mm and 72.35°, and the sensitivity and specificity for MA were 75.6% and 86.5%, respectively. The AUCs of the blood coagulation parameters FIB and D‐D were 0.796 and 0.726, with optimal cutoff value of 4.58 g/L and 0.76 mg/L, respectively. Furthermore, the specificity of FIB was 91.1%, When MA_thrombin_ ≥ 69.35 mm, α‐angle ≥ 72.35° and FIB ≥ 4.58 g/L, patients were in a hypercoagulable state with aggravated thrombosis, which has considerable application value in evaluating the surgical treatment of LEASO (*p* < 0.001).

In conclusion, this retrospective study evaluated elderly patients with LEASO. Compared with D‐D and FIB, TEG results consistently reflected coagulation state, thus confirming a significant correlation between TEG and coagulation function. Elevated coagulation FIB and D‐D levels can be combined with increased MA and α‐angle of TEG to determine systemic hypercoagulation status and thrombosis risks. Its clinical significance lies in early warning of hypercoagulability and thrombosis caused by vascular endothelial cell injury. When the MA, α‐angle, and FIB values were higher, the patient is at higher risk and should be treated with surgical therapy. Postsurgery, if the TEG parameters reduced, the surgery was relatively successful; otherwise, a risk of further blockage exists. TEG requires only a small amount of intravenous whole blood, which reduces the diagnostic reporting time and can help clinicians evaluate disease severity in a timely and accurate manner. The application of TEG and coagulation indices provides a more rapid and intuitive supplementary means for early monitoring of LEASO, reflects the degree of lesions, and provides a new perspective for evaluating surgical treatment.

This study had some limitations. First, it was based solely on data from a single institution within the same country, necessitating the inclusion of data from multiple hospitals to enhance reliability. Another limitation pertained to incomplete follow‐up data, despite our attempts to obtain comprehensive patient information. To better understand the generalizability of our conclusions for elderly patients diagnosed with LEASO, further studies should be conducted in patients with different clinical symptoms.

## Author Contributions

Z.H., P.W. and L.X. designed the study, Z.H., Z.F. and X.W. participated in the collection and analyzed data, Z.H. wrote and prepared the original draft, Z.H., X.W. and X.B. confirm the authenticity of all the raw data. All authors read and approved the final version of the manuscript.

## Ethics Statement

This study was conducted in accordance with the Declaration of Helsinki (2008) and approved by the Ethics Committee of Liyuan Hospital, Tongji Medical College, Huazhong University of Science and Technology [2023], IEC (RYJ002). All procedures were in accordance with the ethical standards of Liyuan Hospital, Tongji Medical College, Huazhong University of Science and Technology Medical Institutional Review Board. Due to the retrospective nature of this study, without the participation of researchers, and the anonymized data of all patients, the Ethics Committee of Liyuan Hospital, Tongji Medical College, Huazhong University of Science and Technology waived the requirement of informed consent, and did not involve personal privacy or commercial interests. All methods were performed in accordance with the relevant guidelines and regulations.

## Consent

The authors have nothing to report.

## Conflicts of Interest

The authors declare no conflicts of interest.

## Data Availability

The data generated in the present study may be requested from the corresponding author.
